# A Treatise on the Diseases and Surgical Operations of the Mouth, and Parts Adjacent: With Notes of Interesting Cases, Ancient and Modern

**Published:** 1851-02

**Authors:** 


*A Treatise on the Diseases and Surgical Operations of the* MOUTH, *and parts adjacent: with Notes of Interesting Cases, ancient and modern.* By M. JOURDAIN, Dentist and Member of the College of Surgery. *Translated from the last French Edition.* Philadelphia, Lindsay & Blakiston, 1851.

This is the title of a royal octavo volume of 444 pages, no less creditable as an increase to the literature of American Dental Surgery, than liberal and tasteful in the mechanical getting up of the book.

Mr. Jourdain's work embraces, in its minute details, both as regards the diseases of which it treats, and the careful report of numerous illustrating cases, a valuable and interesting amount of knowledge to the dentist, as also to the medical practitioner. It is one peculiarly needed, inasmuch as its matter is for the most part confined to the disturbances arising *from* the teeth, rather than *in* them, and involves a correct knowledge of the general principles of surgery and pathology, too frequently overlooked in treating of *specialities*, and not less in the management of an unhealthy mouth.

The translator of the work has modestly omitted giving his name to the public, and hence, perhaps, we may with the greater freedom commend the talent and good judgment with which he has performed his task, not merely rendering the thoughts of the French writer into good English, but—*currente calamo*—reviewing or correcting his errors in the most important feature of the work, with such discrimination as to preclude the necessity of that duty being attempted after him. And yet, the book in its present English dress, has claims to our notice not less than to our thanks, for it is a better work than the original, and especially where the translator has made most free in pruning and adapting "*by suitable additions and corrections to the present wants of the dental profession.*"

Mr. Jourdain, however, gave evidences of extraordinary industry and minute observation of the anatomy and sympathetic organization of the mouth ; and especially, considering the period at which he wrote, (about 1770,) his work must be regarded as evincing scientific views of pathology and hygiene, as applicable to the exercise of his profession, far in advance of his contemporaries. The treatment of his reported cases, proves him to have been a surgeon in whose practice as a *Dentist* we might now properly condemn the want of a due estimate of the value of the teeth, if we may judge, for instance, by the governing rule suggested in a case of tumor, where he was not suffered to operate, but where he states, that if allowed, "*I should have removed all the teeth, good and bad*, involved in the tumor."—There is great probability that the sacrifice of the *bad* ones would have proved that they alone were so involved.

With great and commendable frankness, Mr. Jourdain acknowledges that he has freely selected from the experience of previous writers, and "after all," that it is not so easy a duty as some persons think, to "make books *from books*": had he said not so easy to make *good* books from *bad* ones, the remark would have been still more correct and appropriate. But his readers of the present day, whilst giving him credit for the research and honest confession of the use made of it, would have preferred a more original work from a man evidently capable of deep thought and sound investigation.

We may safely recommend the volume as worthy of both purchase and perusal.

